# Chemometrics in Tandem with Hyperspectral Imaging for Detecting Authentication of Raw and Cooked Mutton Rolls

**DOI:** 10.3390/foods10092127

**Published:** 2021-09-09

**Authors:** Hongzhe Jiang, Yi Yang, Minghong Shi

**Affiliations:** 1 College of Mechanical and Electronic Engineering, Nanjing Forestry University, Nanjing 210037, China; mhshi@njfu.edu.cn; 2 Beijing Research Center of Intelligent Equipment for Agriculture, Beijing 100097, China; yangyi@nercita.org.cn; 3 National Research Center of Intelligent Equipment for Agriculture, Beijing 100097, China

**Keywords:** hyperspectral imaging, meat rolls, chemometrics, visualization, meats substitution

## Abstract

Authentication assurance of meat or meat products is critical in the meat industry. Various methods including DNA- or protein-based techniques are accurate for assessing meat authenticity, however, they are destructive, expensive, or laborious. This study explores the feasibility of chemometrics in tandem with hyperspectral imaging (HSI) for identifying raw and cooked mutton rolls substitution by pork and duck rolls. Raw or cooked samples (*n* = 180) of three meat species were prepared to collect hyperspectral images in range of 400–1000 nm. Spectra were extracted from representative regions of interest (ROIs), and spectral principal component analysis (PCA) revealed that PC_1_ and PC_2_ were effective for the identification. Different methods including standard normal variable (SNV), first and second derivatives, and normalization were individually employed for spectral preprocessing, and modeling methods of partial least squares-discriminant analysis (PLS-DA) and support vector machines (SVM) were also individually applied to develop classification models for both the raw and the cooked. Results showed that PLS-DA model developed by raw spectra presented the highest 100% correct classification rate (CCR) of success in all sets. After that, effective wavelengths selected by successive projections algorithm (SPA) built optimal simplified models which didn’t influence the modeling results compared with full spectra regardless of the meat roll states. Therefore, SPA-PLS-DA models were subsequently used to visualize the raw and cooked meat rolls classification. As a consequence, the general meat species of both raw and cooked meat rolls were readily discernible in pixel-wise manner by generating classification maps. The results showed that HSI combined with chemometrics can be used to identify the authentication of raw and cooked mutton rolls substituted by pork and duck rolls accurately. This promising methodology provides a reference which can be extended to the classification or grading of other meat rolls.

## 1. Introduction

The global production and consumption of meats are expected to continue to grow steadily in the next decade [1]. As a result, the analysis of quality and safety related issues is attracting global concerns which will significantly influence the consumers’ choices to buy or re-buy. Driven by profits, intentional meat adulteration or meat fraud conducted by unscrupulous businessmen frequently occurred which substituted valuable species by cheap cuts [2]. For examples, the horsemeat scandal in 2013 and spoiled meat scandal in 2017 caused huge panic [3]. These cases raise a number of issues related to economy (e.g., earn more money), religion (e.g., pork for Muslims), diet (e.g., calories), and lifestyle (e.g., vegetarianism) [4]. Therefore, the detection of meat authentication is indispensable not only to consumers but also to the meat industry.

Mutton is one of the most popular meats, mainly due to its unique flavor, rich nutrition, and benefits for health. Mutton roll, or known as mutton slice, is the major ingredient in Chinese hotpot and is very popular in China. Meat rolls of different meat species are similar in appearance, which makes it a vulnerable target for profit-driven fraud. In fact, the undeclared replacement of mutton in meat rolls with low-priced meats such as pork and duck frequently occurs [5]. Therefore, the detection of mutton roll authentication using a non-invasive, rapid, and practical method is of vital importance to protect consumers’ rights. Generally, the meat species of raw meat roll can be hardly identified only by the naked eye. In fact, fake cooked meat roll is more difficult to be detected than raw meat roll due to the radical changes related to structure, protein, and DNA during the cooking procedure [6].

Currently, numerous analytical methods have been proven to be effective for meat species identification. Among them, techniques based on polymerase chain reaction (PCR) [7], enzyme-linked immunosorbent assay (ELISA) [8], high-performance liquid chromatography (HPLC) [9], loop-mediated isothermal amplification (LAMP) [10], immune strip test [11], and mass spectrometry [12], are available in previously published reports. These methods were found to be sensitive, but required stationary laboratories, high-tech equipment, and sophisticated sample preparation such as DNA, proteins, or organic compounds extraction. The fact is that they have different limitations of being difficult for result interpretation, time-consuming, laborious, or expensive. Additionally, a few chemical-free alternative techniques have been applied to replace instrumental methods for assessing meat adulteration or meat fraud. Near-infrared spectroscopy (NIRS) is advantageous in rapid, simple, non-destructive, and multicomponent simultaneous detection of samples [13,14,15]. Over the last decade, NIRS has been successfully proven to be an effective tool in detecting meat adulteration or meat fraud [16]. In addition, several studies have also been conducted on meat adulteration in cooked samples [17,18]. However, the detection accuracy of this spectroscopic technique is limited by the single-point detection which could hardly represent the whole sample.

The hyperspectral imaging (HSI) technique has recently gained attention in a plethora of research areas including food and agricultural products [19,20]. The simultaneous acquirement of spatial and spectral information makes HSI more competitive than traditional imaging or spectroscopic techniques. The most prominent advantage is that it could intuitively distribute internal and external quality and safety attributes. In recent years, HSI has been employed in widespread applications in meat and meat products. Related to meat adulteration identification, previous studies focused on detecting carrageenan adulteration in minced chicken [21], pork adulteration in minced beef [22], duck adulteration in minced lamb [23], horsemeat adulteration in minced beef [24], jowl meat adulteration in minced pork [25], etc. To our knowledge, no endeavors have been made to identify raw or cooked meat roll authentication.

Therefore, the purpose of this study was to analyze the feasibility of visible and near-infrared (VNIR, 400–1000 nm) HSI combined with chemometrics to detect and visualize authentication of raw and cooked mutton rolls. The specific aims of this research were to (1) collect the VNIR hyperspectral images of mutton, pork, and duck meat rolls in both raw and cooked states; (2) identify the characteristic regions of interest (ROIs) from hyperspectral images, and extract representative spectral information; (3) carry out principal component analysis (PCA) to seek the preliminary spectral distinguishability; (4) compare the effects of four different spectral preprocessings on established partial least square-discriminant analysis (PLS-DA) and radial basis function support vector machine (RBF-SVM) models; (5) identify effective wavelengths for classifying raw and cooked meat rolls of different meat species, respectively; (6) compare the performances of simplified models based on selected wavelengths, and choose the optimal ones; (7) construct the classification maps using the optimal simplified models to visualize the overall identification results.

## 2. Materials and Methods

### 2.1. Sample Preparation

Fresh mutton, pork and duck were purchased from a Suguo supermarket located in Nanjing, China in September, 2020. Different meats with layers of fat and lean meat in it were sold at 0 to 4 °C under refrigeration. The mutton (*longissimus* thoracis), pork (*M. longissimus* dorsi) and duck (thoracis) meat were purchased, packed separately using a portable refrigerated incubator for meat preservation, and transported to our laboratory within 8 min. Afterward, visible connective tissues were first removed, and then meat samples were cut into long column blocks with a kitchen chopper. The size of stacked meat blocks was about 150 mm × 70 mm × 70 mm (length × width × height), and then samples were shaped to long meat towers using food cling wrap film and a fixed cylinder with the cross-sectional area diameter of 80 mm. Finally, three meat towers including pure mutton, pure pork, and pure duck were individually prepared. All the meat towers were frozen at −15 °C in refrigerator for a week. The used tools were carefully washed with hot water and detergent during different meat cuttings.

In the sampling days, frozen meat towers were taken out and cut using a hand-mincing meat slicer (XT0019, Xinbai Co. Ltd., Fuyang, Anhui, China). The thickness parameter of the slicer was set to 2 mm. As a result, a total of 180 2-mm-thick meat roll slices (60 pure mutton rolls, 60 pure pork rolls and 60 pure duck rolls) were prepared. Each meat roll was placed in a round empty Petri dishes (90 mm in diameter × 4 mm deep), respectively. Raw hyperspectral images were then collected using our HSI system. After images collection, each meat rolls of the abovementioned raw samples were cooked in boiling water in a pot at 100 °C for 1 min until no visible raw areas could be observed in the samples. Then cooked meat rolls were cooled at room temperature, and paper towel was used to remove the surface liquid. At last, hyperspectral images of all the cooked rolls were also captured. Regardless of the samples were in raw or cooked states, a total of 120 samples (3 categories × 40 samples per category) were employed for calibration or cross-validation set, while the residual 60 samples (3 categories × 20 samples per category) were applied for independent prediction set.

### 2.2. Hyperspectral Imaging Protocol

In this research, prepared meat rolls were scanned using a push-broom line-scanning HSI system in range of 380.6 nm to 1012.2 nm in reflectance mode. This HSI system consisted of a spectrograph (ImSpector V10E, Spectral Imaging Ltd., Oulu, Finland), a charge-coupled device (CCD) camera (12-bit), a moving stage driven by a stepping motor (SC30021A, Zolix, Instrument Co., Beijing, China), a uniform illumination unit with a pair of 150 W tungsten-halogen lamps, a computer (Lenovo Tianyi 510 Pro, Lenovo Group Ltd., Beijing, China), and the connected software (Isuzu Optics Corp., Taiwan, China). Other details and parameters related to the system could be further found in Jiang et al. [26].

Consequently, hyperspectral images containing 300 channels with the spectral resolution of 2.8 nm were collected. In order to reduce the effects of dark current, all the acquired raw hyperspectral images were then calibrated using white and dark reference images. White reference image was saved by scanning a Teflon white board (about 99.9% reflectance). Dark reference image was scanned by covering lens with black cap (about 0% reflectance). Then, the calibration procedure was conducted using the following formula:

(1) $$I_{c}{=(I}_{o}-\;D)/(W\;-\;D)\times 100\%$$

where *I*_c_ and *I*_o_ were the calibrated and raw hyperspectral image, *D* denoted the dark reference image, and *W* indicated the white reference image.

### 2.3. Image Segmentation and ROIs Identification

**Figure 1:**
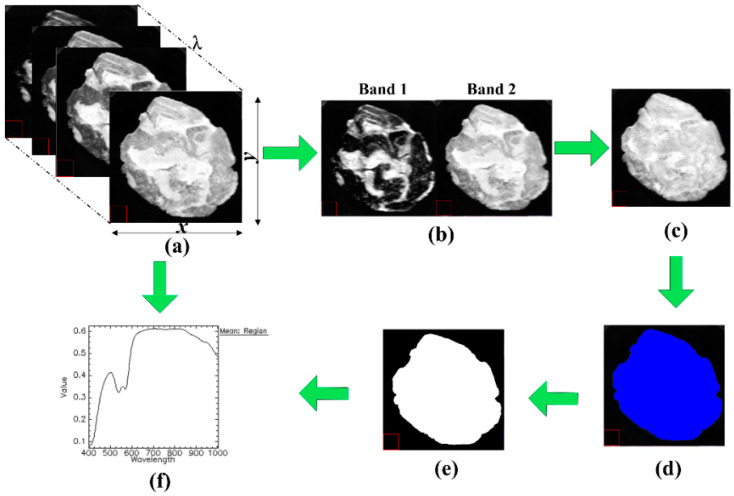
Steps for image segmentation. (**a**) calibrated hyperspectral images; (**b**) band images with low and high reflectance intensity (415 nm and 800 nm); (**c**) resulted image using band math function; (**d**) ROI identification by thresholding; (**e**) mask establishment; and (**f**) spectra extraction.

Image segmentation is one of the key steps in hyperspectral image preprocessings, which might significantly influence the effectiveness of subsequent extracted spectra. The main procedures of image segmentation and spectra extraction from hyperspectral images are depicted in Figure 1. All the involved steps were conducted using the functions in ENVI 5.1 (Research Systems Inc., Solutions, Boulder, CO, USA). The calibrated hyperspectral image consists of a series of two-dimensional images and is shown in Figure 1a. Among them, two characteristic images with the highest and lowest reflectance intensity were chosen (415 nm and 800 nm, Figure 1b). The resulting greyscale image in Figure 1c was constructed by subtracting image at 415 nm (band 1) from image at 800 nm (band 2) using band math function in ENVI software. The background was then excluded by applying a fixed threshold of 0.15 to Figure 1c, producing a segmented region in blue named ROI in Figure 1d which includes the whole meat roll part only. The isolated ROI was treated to produce a binary mask (Figure 1e), and the mask was then applied back to raw calibrated hyperspectral images to be further used in extracting spectra (Figure 1f). Therefore, a mean reflectance spectrum for each sample was extracted by averaging the spectral responses of all retained pixels in the masked images.

### 2.4. Exploratory Data Analysis

PCA is one of the most commonly used unsupervised chemometric tool for exploring hidden information in spectral data. PCA first transforms original spectral variables to several linear independent variables named principal components (PCs) [27]. After that, the co-linearity in the dataset was reduced by selecting the first few effective PCs [28]. The scores of selected PCs can be applied to create score plots to demonstrated spectral relationships among different samples. In score plots, data points in the same category will cluster closely. Additionally, the corresponding selected PC loadings will also be drawn, weighted peaks and valleys of which are useful in extracting characteristic wavelengths. In our study, PCA was conducted to seek the distinguishability of mutton, pork, and duck rolls based on extracted spectra.

### 2.5. Spectral Preprocessing

To effectively reduce the influence of noise and background caused by environmental and instruments’ interference, preprocessing of the extracted spectra before subsequent analysis was implemented. The standard normal variable (SNV) was used to convert each spectrum into a zero average intensity value of unit standard deviation to eliminate the deviation caused by particle size and scattering [29]. Normalization was employed using minimum maximum normalization method to eliminate multiplicative spectral effects by converting the spectral vector into a unit length [30]. First and second order (1st and 2nd) derivatives were applied to eliminate non-chemical effect and resolve problems of overlapping bands by correcting the baseline effect in the spectrum. In our study, 1st and 2nd derivatives were based on Savitzky-Golay smoothing of second-order polynomial fit with a five-point moving window.

### 2.6. Wavelengths Selection Methods

Given that high-dimensional spectra contained redundant collinear information, effective wavelengths selection was necessary to reduce model complexity [31]. Simulated annealing algorithm (SAA) is a probabilistic global optimization method based on the physical annealing process of solids. During optimization, SAA allows acceptance of an inferior solution, and could traverse local optimums and find the global optimal solution. In SAA, the problem starts with an initial solution, which is modified iteratively according to a control parameter *T* (similar with temperature). As the parameter *T* decreases, the convergence criterion becomes more and more difficult to satisfy. Finally, if *T* is low enough, there can be no further change in the solution space. More details of SAA could be found in the literature [32]. In this study, the initial temperature was set as 50, and reduction rate was set as 0.96.

Random frog (RF) coupled with PLS is an efficient variable selection method to assess the importance of features, which possesses the advantages of reversible jump Markov Chain Monte Carlo [33]. RF algorithm works iteratively to calculate the selection probability of each variable. The involved basic steps are, (1) a variable subset is randomly generated from the original variables as the initial subset; (2) a new candidate subset is randomly generated based on the initialization subset, and the initialization subset is updated with the new candidate subset with a certain probability; (3) the selection probability of each variable can be calculated after predefined *N* iterations of step 2 [34]. In this study, the number of iterations was set as a sufficient amount of 1000, and the wavelengths with higher selection probability were likely selected as sensitive variables.

Successive projections algorithm (SPA) is a wavelength selection method that aims to solve the problem of colinearity by choosing wavelengths with the least redundancy [35]. For this reason, SPA uses a simple projection operation in the vector space to find a small representative set of spectral variables with a minimum level of collinearity. The variable selection principle of SPA is to construct a candidate subset of *N* variables based on a series of projection operations involving the instrument response matrix column [36]. The range of *N* parameter was set from 1 to 10, and then certain appropriate wavelengths were retained and selected.

### 2.7. Model Development and Assessment

Partial least square-discriminant analysis (PLD-DA) is a commonly used qualitative modeling method in spectroscopy to maximize the correlation between spectra and aimed categories [37]. The PLS-DA model seeks to correlate spectra (*X*-variables) with the defined classes (*Y*-variables), attempting to maximize the covariance among the spectra of different meat species. In this study, the *Y*-variable assigned for the mutton roll class was “1”, pork roll class was “2”, and duck roll class was “3”. The optimal number of latent variables (LVs) was determined using leave-one-out cross-validation (LOOCV) with “venetian blinds” method in the calibration set. The number of LVs for the lowest error rate (misclassification) was finally chosen to build the optimal PLS-DA model.

Support vector machine (SVM) is a supervised classification algorithm which is also widely used in spectral analysis. SVM classifies samples by exploring a hyperplane to segment nonlinear spectral data of prepared samples [38]. The radial basis function (RBF) has strong ability in addressing nonlinear problems, which was thus used as the kernel function in this research. The parameters in regularization *c* and kernel function *g* for RBF-SVM model were optimized in grid-search process. The values of *c* and *g* for searching were set from 2^−8^ to 2^8^. The RBF-SVM model with the highest assessment indicators was deemed as the optimal RBF-SVM model and selected for further analysis.

To determine the work of the above established qualitative models, correct classification rate (CCR) in calibration, cross-validation and prediction sets were individually evaluated using the following equation:

(2) $$\mathrm{CCR}=\frac{N_{1}}{N_{2}}\times 100\%$$

where CCR indicates correct classification rate, N_1_ is the number of precisely sorted samples in calibration, cross-validation or prediction set, and N_2_ represents the corresponding total number of samples in calibration or prediction set.

Confusion matrix was utilized to sum up the classification performance of the models. The true value and false value were individually represented by diagonal or off-diagonal lines in the matrix. The performances were evaluated using three effective statistical parameters, i.e., the sensitivity, specificity and efficiency. Among them, sensitivity (SEN) describes the model’s ability in accurately recognizing samples to the corrected class. Specificity (SPE) expresses the model’s ability in correctly rejecting samples to the uncorrected classes. Efficiency (EFF) indicates the correct identification probability of the samples’ classes by calculating the geometric mean of sensitivity and specificity [39]. The definitions of sensitivity, specificity and efficiency for confusion matrix were also calculated as below:

(3) $$\mathrm{SEN}=\frac{\mathrm{TP}}{\mathrm{TP}+\mathrm{FN}}$$

(4) $$\mathrm{SPE}=\frac{\mathrm{TN}}{\mathrm{TN}+\mathrm{FP}}$$

(5) $$\mathrm{EFF}=\sqrt{\mathrm{Sensitivity}\times \mathrm{Specificity}}$$

where TP represents the true positive, TN means the true negative, FN denotes the false negative, and FP indicates the false positive.

Kappa coefficient is also an effective index to indicate the differences between reference and predicted categories in the confusion matrix and was also calculated in this study. It. Kappa coefficient is based on the agreement of classifications and the number of times the samples were distributed in the same class. This value ranges from 0 to 1, and the “1” means perfect agreement while “0” means on agreement. The higher the Kappa coefficient is, the more robust the model is. Kappa coefficient is defined using the following equations:

(6) $$\mathrm{Kappa}\;\mathrm{coefficient}=\frac{P_{o}-P_{e}}{1-P_{e}}$$

(7) $$P_{o}=\frac{\mathrm{TP}+\mathrm{TN}}{\mathrm{TP}+\mathrm{FN}+\mathrm{TN}+\mathrm{FP}}$$

(8) $$P_{e}=\frac{\sum_{1}^{M}\left[{\left(\mathrm{TP}+\mathrm{FN})*(\mathrm{TP}+\mathrm{FP}\right)}_{i}{+(\mathrm{FP}+\mathrm{TN})*(\mathrm{TN}+\mathrm{TN})}_{i}\right]}{{\left(\mathrm{TP}+\mathrm{FN}+\mathrm{TN}+\mathrm{FP}\right)}^{2}}$$

where *M* indicates the total number of categories, *i* represents the number of categories.

Receiver operating characteristics (ROC) curves were often depicted to visualize and assess the performance of models [40]. Instead of fixing a constant threshold for predicted scores, the rate of false positives (FP) was drawn against the rate of true positives (TP) for each possible threshold value using a graph. In the graph, AUC indicated a portion of the area so that the value would always lie between 0 and 1. An excellent model usually provides a big value of the area under the ROC curve (AUC) [41]. A model with AUC value of 0.5 to 1 expressed that a good classifier was acquired. Besides, model with AUC lower than 0.5 meant this classifier failed. In our research, the parameters of Kappa coefficient and AUC were both calculated using a homemade program.

### 2.8. Classification Map Visualization

The visualization step is beneficial to understand the general situation of the samples. Since each pixel in a hyperspectral image has its own spectrum, the meat species for each pixel can be calculated and visualized using a classification map [42]. Generally, the three-dimensional (3D) multispectral image (hyperspectral image at selected wavelengths) was primarily spread to a two-dimensional (2D) matrix, and then calculated the classes by applying back a preferred model based on characteristic wavelengths. After that, the resultant matrix was folded back to generate a classification image. As is known that the precise meat species determination of all the pixels within one whole roll is difficult, the pixels belong to the same meat species would appear in the same color so that the meat species of all spots were easily interpretable.

## 3. Results and Discussion

### 3.1. Spectral Overview for the Raw and Cooked Meat Rolls

**Figure 2:**
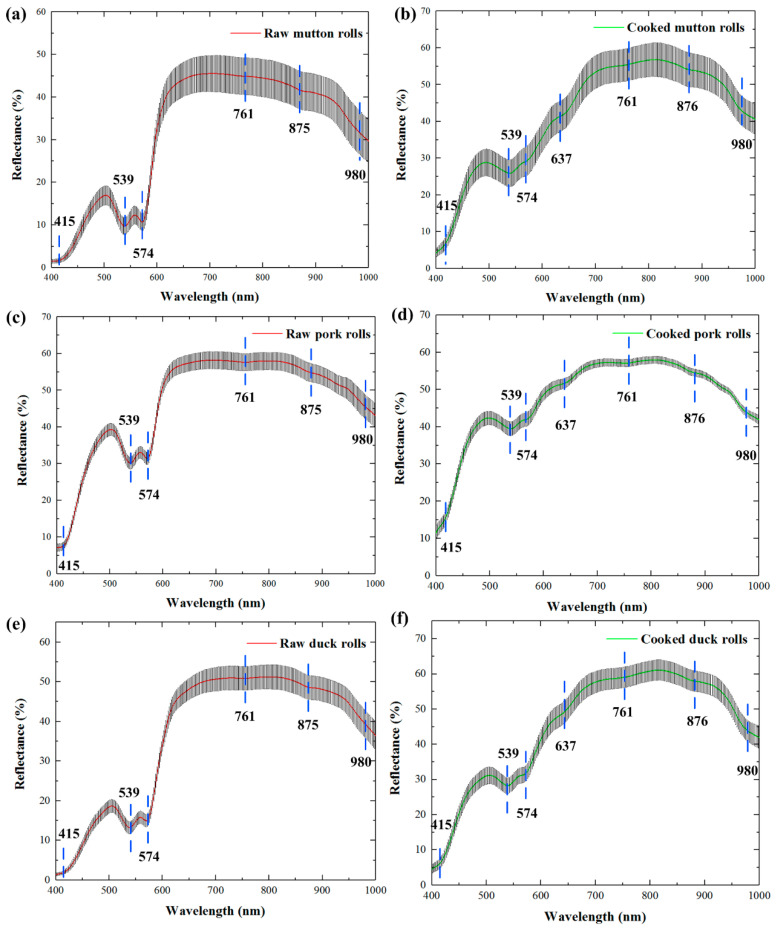
The average reflectance spectra with standard deviation (SD) for raw and cooked meat rolls of mutton (**a**,**b**), pork (**c**,**d**), and duck (**e**,**f**).

Due to the attenuation of the CCD response, two low signal-to-noise spectral regions (380–400 nm and 1000–1012 nm) were excluded. Thus, the extracted spectra from hyperspectral images were collected over a spectral range of 400–1000 nm (285 wavelengths). Figure 2 shows the average spectra obtained from raw and cooked meat rolls (i.e., mutton, pork and duck) along with standard deviation (SD) of individual spectra from the mean. The three species of meat rolls showed similar profiles individually for the raw and the cooked, which could be derived from their similar tissue compositions, structures, and color presentation. For the visible band in range of about 400–650 nm, the reflectance was low especially for the raw meat rolls. The spectral reflectance of about 650–850 nm began to rise and maintained at a high level. From about 850 nm to 1000 nm, the spectral reflectance continued to descend. Through the comparison of spectra between raw and cooked meat rolls, the reflectance differences were relatively larger in range of 650–1000 nm than in 400–650 nm. The spectra of pork rolls (Figure 2c,d) displayed less variance with respect to those of mutton rolls (Figure 2a,b) and duck rolls (Figure 2e,f). In addition, cooked meat rolls showed higher reflectance levels than raw meat rolls, which was mainly due to the water leakage during cooking [18].

In detail, it could also be observed that several downwards reflectance valleys (i.e., absorbance peaks) existed, which reflected prominent spectral features in Figure 2. The wavelength at 415 nm corresponded to the Soret absorption band which was related to the respiratory pigment of haemoglobin [43]. The reflectance valleys at 539 nm and 574 nm were individually ascribed to the contents of deoxymyoglobin and oxymyoglobin representing meat color traits [44]. The absorbance bands at around 761 nm and 980 nm were associated with the third and second stretching O-H overtone of water, respectively [45]. A weak reflectance valley of 875/876 nm related to the C-H stretching vibration from aliphatic compounds [46]. For cooked meat rolls, another weak valley of 637 nm was more evident than the raw, which appeared to be related to methemoglobin absorption regions [47]. These characteristics of the intrinsic differences combined with effective chemometrics methods provided the likelihood for identifying different meat roll samples.

### 3.2. Principal Component Analysis Results

**Figure 3:**
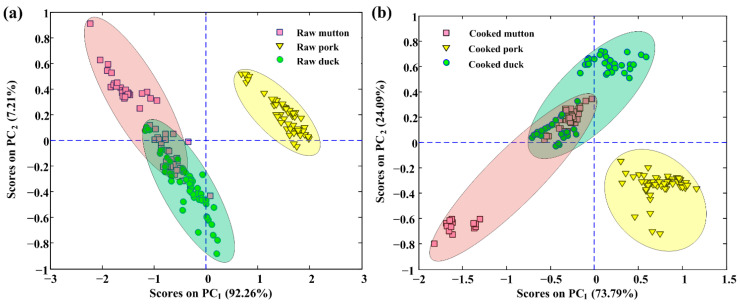
Score plots for the first two PCs (PC_1_ vs. PC_2_) using spectra collected from (**a**) raw and (**b**) cooked meat rolls.

Considering the high dimensionality of extracted spectra, redundant information is inevitable. To solve this problem, PCA was applied to explore the spectral differences and examine the natural pattern among samples in more detail. The calculated PCA results showed that the first two PCs can express the cumulative spectral variance of 99.47% (PC_1_ accounts for 92.26%, and PC_2_ accounts for 7.21%) for spectra of raw meat rolls. As for spectra of cooked meat rolls, the first two PCs accounted for a total variance of 97.88% (PC_1_ accounts for 73.79%, and PC_2_ accounts for 24.09%). The score plots (PC_1_ vs. PC_2_) are reported in Figure 3 (Figure 3a is for raw meat rolls, and Figure 3b is for the cooked), ellipses are drawn herein to denote their covered regions to intuitively see the distribution. The clusters of these three classes were generally separated by the first two PCs although the overlapping between mutton and duck classes existed. The variability was probably due to the high fingerprint similarity of organics in mutton and duck. In addition, pork rolls were perfectly clustered into one group without any overlap with other groups. The observation indicated that pork was quite different from mutton and duck in chemical composition, mainly including fat content. The score plots have demonstrated the separability among the meat rolls of three different meat species, however, the application of spectra alone was difficult to perfectly discriminate them no matter meat rolls were raw or cooked, so it was expected that the further use of chemometrics and modeling methods would work in this discrimination.

**Figure 4:**
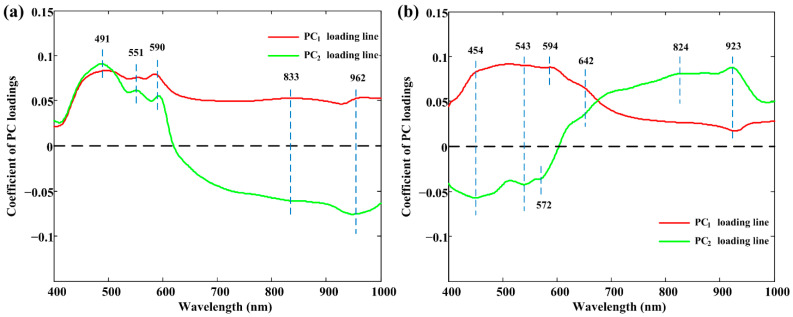
The PC_1_ and PC_2_ loading lines for spectra collected from (**a**) raw and (**b**) cooked meat rolls. PC: principal component.

In more detail, Figure 4 shows the loading lines of these two PCs in PC spaces. Peaks and valleys with high absolute coefficients were deemed to be effective in classifying different classes. This comprehensive analysis showed that wavelengths centered at 491 nm, 551 nm, 590 nm, 833 nm, and 962 nm can be selected as effective wavelengths for raw meat rolls. As for cooked meat rolls, 454 nm, 543 nm, 572 nm, 594 nm, 642 nm, 824 nm, and 923 nm were retained. Some wavelengths were consistent with the ones selected in spectral properties analysis in Section 3.1. These important wavelengths were also attempted in the simplified models’ construction.

### 3.3. Identification of Mutton Rolls with Full Spectra

Prior to modeling, spectra were first preprocessed with SNV, normalization, 1st, and 2nd derivatives to enhance the useful information. In order to correctly identify raw and cooked mutton rolls, PLS-DA and RBF-SVM were applied to raw and preprocessed spectra to develop classification models, respectively (Table 1). The PLS-DA and RBF-SVM approaches provided excellent classification results with the CCRs in calibration, cross-validation, and prediction sets ranged from 80% to 100%. For raw meat rolls, PLS-DA model showed slightly better classification results than RBF-SVM, with CCRs of 100% in all the three sets. As for cooked meat rolls, RBF-SVM models also performed worse than PLS-DA models by showing CCRs in prediction set of 95.0% to 100% compared with 98.3% to 100%. Through comparison, a more appropriate modeling method of PLS-DA was finally chosen for further evaluation.

Considering the various preprocessing methods in PLS-DA models development, SNV, normalization, 1st, and 2nd derivatives didn’t improve or even reduced the classification accuracy. Raw spectra performed considerably well that CCRs = 100% in all sets regardless of modeling methods and the states of meat rolls. These results implied that raw spectra were informative enough and any extra spectral preprocessings done to the raw spectral data was not necessary. As a result, raw spectra with PLS-DA modeling methods were chosen and would be employed in the subsequent analysis.

### 3.4. Effective Wavelengths Selection

The selection of individual wavelengths related to meat species is highly desired. Since effective wavelengths constitute a significant procedure in further developing portable devices, wavelengths selection plays an essential subject in the spectral field of meat rolls identification [48]. For this reason, wavelengths selection is helpful in the sense that the few effective wavelengths simplify the model by avoiding redundant information. Different variable selection algorithms including RF, SAA, and SPA were individually applied to identify featured wavelengths for raw and cooked meat rolls, and all the selected wavelengths are displayed in Table 2. The number of wavelengths selected for raw and cooked meat rolls was not more than 8. Whether raw or cooked meat rolls, SPA selected the least optimal wavelengths, while RF selected the most. After this process, the number of wavelengths was significantly reduced by at least 97.2% to at most 98.9%. Nevertheless, it is important to further evaluate the results of simplified models developed by these selected sets of spectral variables. Although the number of wavelengths decreased, those potential variables significantly reduced the accuracy of models would not be considered.

### 3.5. Simplified Models Based on Selected Wavelengths

The main statistics in calibration, cross-validation, and prediction sets obtained by the PLS-DA models based on various selected wavelengths are shown in Table 3. All these simplified models yielded good results which illustrated that the selected wavelengths can be used to elucidate the meat species in raw and cooked meat rolls. For raw meat rolls, the RF-PLS-DA and SAA-PLS-DA models showed satisfactory results with CCRs of 93.3% and 95.0% in prediction set. Furthermore, wavelengths selected by SPA and PC loadings didn’t influence the modeling results compared with full spectra but speeded up the modeling procedure. With regard to cooked meat rolls, SPA-PLS-DA also performed the best, while RF, SAA and PC loadings selected wavelengths performed not as good as full spectra. The assessment indicators also exhibited high level of discriminant ability with SEN = 1.00, SPE ≥ 0.90, EFF ≥ 0.95, AUC ≥ 0.85, and Kappa coefficient ≥ 0.90 for the raw meat rolls identification. As for cooked meat rolls, the calculation of these values showed SEN ≥ 0.65, SPE = 1.00, EFF ≥ 0.81, AUC ≥ 0.91, and Kappa coefficient ≥ 0.83 in prediction set.

**Figure 5:**
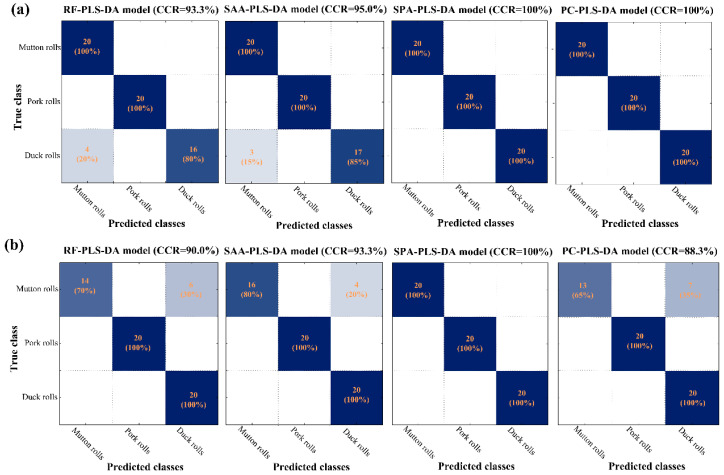
Confusion matrices in prediction set of PLS-DA models developed by various methods-selected wavelengths for (**a**) raw and (**b**) cooked meat rolls classification. RF: random frog; SAA: simulated annealing algorithm; SPA, successive projections algorithm; PC, principal component; PLS-DA: partial least squares-discriminant analysis.

Figure 5 presents the detailed confusion matrices produced by the developed models in the external prediction set. Good performances could be observed for all the models, with the CCR in prediction set over 88%. The left label showed the true classes while the bottom label indicated the classes predicted by the various simplified models. Values in the main diagonal line meant the correctly classified samples while the values outside the main diagonal indicated the misclassified samples. In Figure 5a, it could be seen that raw duck rolls were sometimes misclassified into mutton rolls when RF-PLS-DA and SAA-PLS-DA models were employed. SPA-PLS-DA and PC-PLS-DA model both showed the best results of CCR = 100%.

As for cooked meat rolls (Figure 5b), misclassification also happened between mutton and duck rolls. The cooked mutton rolls were likely to be misclassified as cooked duck rolls. The above misclassification of raw and cooked samples matched the PCA spectral analysis results in Figure 3. The RF-PLS-DA, SAA-PLS-DA, and PC-PLS-DA models failed to achieve the 100% CCRs in prediction set. The SPA-PLS-DA model performed best with CCRs of 100% in all datasets. Overall, SPA-PLS-DA not only maintained the predicted accuracy but also reduced the complexity. The performance demonstrated that SPA was the most suitable method in selecting featured wavelengths regardless of the raw or cooked meat states. As a result, SPA-selected wavelengths (404 nm, 578 nm, 598 nm, and 936 nm for the raw, and 400 nm, 478 nm, and 652 nm for the cooked) seemed to be effective in identifying both raw and cooked meat rolls and were adopted in subsequent visualization procedure. In addition, the simplification of SPA method will offer the possibility to perform analysis away from the laboratory using an efficient portable, handheld, and micro-optical device.

### 3.6. Classification Visualization

**Figure 6:**
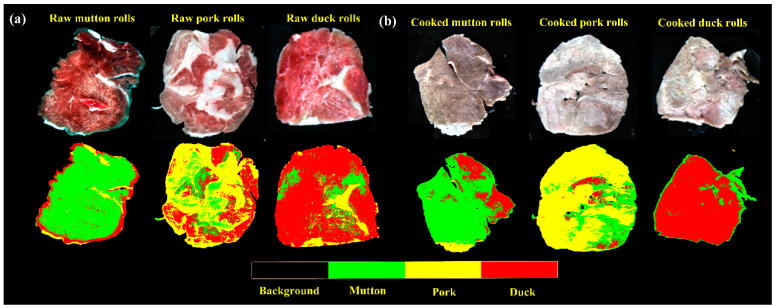
The visualization of classification maps of (**a**) raw and (**b**) cooked meat rolls.

Visualization for HSI is indeed one of the most attractive advantages in food screening. Prior to visualization, each hyperspectral image was resized by selecting those images at SPA-selected wavelengths to form a new multivariate image. After that, the two optimal simplified SPA-PLS-DA models were applied back to multivariate images to individually classify meat species of the raw and cooked rolls. Figure 6 shows the resultant intuitive classification maps with a color bar listed at the bottom. For comparison, the first row showed the pseudo-RGB images combining three wavelengths of 640 nm, 550 nm, and 460 nm into red (R), green (G), and blue (B) channels, respectively. The ones in second row represented the intuitive classification maps. In the classification maps, different color assignments indicated different classified meat species. By using a color bar of limited four colors, meat species including mutton, pork and duck were shown in green, yellow and red, respectively. Additionally, the background was denoted by black color. As shown, the classification of meat species in different spatial spots can be easily visualized regardless of raw (Figure 6a) or cooked (Figure 6b) samples. Composite of different colors in one identical sample indicated that there were some misclassified pixels. However, the general species of meat rolls can be identified by observing most of the predicted classes.

To further make a clear interpretation of Figure 6, statistics of the number of pixels classified into different meat species are shown in Table 4. Regarding the mutton rolls, mutton pixels occupied a most proportion of 79.87% and 75.94% for raw and cooked mutton rolls, respectively. Similarly, for the other two species, pork and duck pixels also gave the highest proportions of 57.99% (raw pork rolls) and 75.01% (cooked pork rolls), 76.07% (raw duck rolls) and 85.86% (cooked duck rolls), respectively. We can also see in Figure 6 and Table 4 that yellow pork pixels have the highest classification error. It seemed that fat composition in intramuscular fat played an indicative role for classifying pork rolls. It was also interesting to find that the observed error for different hyperspectral images of the same sample can vary in a small range. This is probably related to the chemical composition distribution and imaged angles. Therefore, by calculating the percentage of pixels in certain class to identify its general class qualitatively can expect to reduce the overall predicted error.

As a consequence, this general visualization accuracy is adequate in detecting the species of meat rolls in actual inspection process. The detection of meat authentication or meat quality using HSI has been reported as well in recent literatures by combining with chemometrics.

This way, Al-Sarayreh et al. [49] found that combining HSI with deep convolution neural networks modeling method achieved the best performance with a 94.4% overall classification accuracy for red-meat products adulteration. Kamruzzaman et al. [50] used HSI and pattern recognition algorithm for different red meat species classification and yielded 98.67% overall classification accuracy. Fan et al. [51] combined HSI with two-dimensional correlation analysis for determining the biogenic amines in mutton during refrigerate storage, and correlation coefficient of 0.91 in prediction set was acquired. As summarized above, previous studies were mainly focused on raw meat samples, however, samples after cooking were rarely covered. In addition, the performances of simplified models based on selected wavelengths were slightly worse, and the results herein achieved CCRs of 100% in all datasets. Current results suggested that the VNIR HSI had the potential for rapidly and non-destructively detecting mutton rolls substitution by pork and duck meat regardless of their raw or cooked states. The visualization results indicated that HSI in tandem with chemometrics was promising for large-scale visual detection of mutton rolls authenticity in modern meat industry. However, one thing to note was that several studies reported that other meats were also commonly used as adulterants including kangaroo [52], turkey [53], offal [54], and horsemeat [55], etc. The possibility of low-cost adulterants other than pork and duck existed. Additional studies should be carried out to include more low-cost meats to enlarge the dataset so that this technique can be used in real on-line applications.

## 4. Conclusions

Overall, this study presented that HSI coupled with chemometrics had great potential in detecting mutton rolls substitution by pork and duck. This is the first report to demonstrate that HSI technique could well identify authenticity of mutton roll in both raw and cooked states. The proposed image segmentation method made the spectra extracted from hyperspectral images had a good correspondence with samples. Results acquired from different preprocessing, machine learning classifiers, and feature wavelength selection methods provided an intuitive way to compare their results and identify the best methods. Four (404, 578, 598 and 936 nm) and three (400, 478 and 652 nm) SPA-selected wavelengths yielded the best performances of 100% classification in building simplified models. Using these optimal effective wavelengths, low-cost, rapid, and simple multispectral imaging instrument can be designed for real-time substituted mutton rolls detection. Spatial classification map was also a benefit to make a quick visual detection which conventional methods cannot achieve. Further work will focus on considering more meat species of meat rolls in experiments to enlarge the dataset and verify the above findings.

## Figures and Tables

**Table 1:** Model performances for identifying raw and cooked meat rolls based on various preprocessings and modeling methods applied to full spectra, PLS-DA: partial least square-discriminant analysis; RBF-SVM: radial basis function-support vector machine; SNV: standard normal variable; LVs: latent variables.

Modeling Methods	Preprocessing	LVs	Correction Classification Rate		
			Calibration Set	Cross-Validation Set	Prediction Set
PLS-DA (raw)	None	3	100%	100%	100%
	SNV	2	100%	100%	100%
	Normalization	2	100%	100%	100%
	1st derivative	2	100%	100%	100%
	2nd derivative	2	100%	100%	100%
RBF-SVM (raw)	None	/	100%	93.3%	80.0%
	SNV	/	100%	100%	100%
	Normalization	/	100%	100%	100%
	1st derivative	/	100%	100%	100%
	2nd derivative	/	100%	100%	100%
PLS-DA (cooked)	None	4	100%	100%	100%
	SNV	2	100%	100%	100%
	Normalization	2	100%	100%	100%
	1st derivative	2	100%	100%	98.3%
	2nd derivative	2	100%	100%	100%
RBF-SVM (cooked)	None	/	100%	100%	100%
	SNV	/	100%	97.5%	95.0%
	Normalization	/	100%	98.3%	95.0%
	1st derivative	/	100%	100%	95.0%
	2nd derivative	/	100%	100%	100%

**Table 2:** Summary of selected wavelengths using RF, SAA, SPA and PC loadings. RF: random frog; SAA: simulated annealing algorithm; SPA, successive projections algorithm; PC, principal component.

States	Method	Number	Wavelengths (nm)
Raw	RF	8	572, 574, 576, 578, 820, 889, 891, 900
	SAA	5	556, 898, 932, 952, 987
	SPA	4	404, 578, 598, 936
	PC	5	491, 551, 590, 833, 962
Cooked	RF	6	636, 648, 652, 659, 684, 707
	SAA	7	413, 440, 505, 556, 580, 680, 882
	SPA	3	400, 478, 652
	PC	7	454, 543, 572, 594, 642, 824, 923

**Table 3:** Results of PLS-DA models using selected effective wavelengths. LVs: latent variables; CCR: correct classification rate; SEN: sensitivity; SPE: specificity; EFF: efficiency; AUC: area under the receiver operating characteristics curve; Kappa: Kappa coefficient.

States	Method	LVs	Calibration Set CCR	Cross-Validation Set CCR	Prediction Set					
					CCR	SEN	SPE	EFF	AUC	Kappa
Raw	RF	4	100%	100%	93.3%	1.00	0.90	0.95	0.85	0.90
	SAA	4	92.5%	89.2%	95.0%	1.00	0.93	0.96	0.89	0.93
	SPA	3	100%	100%	100%	1.00	1.00	1.00	1.00	1.00
	PC	4	100%	100%	100%	1.00	1.00	1.00	1.00	1.00
Cooked	RF	3	100%	100%	90.0%	0.70	1.00	0.84	0.93	0.85
	SAA	4	100%	100%	93.3%	0.80	1.00	0.89	0.95	0.90
	SPA	3	100%	100%	100%	1.00	1.00	1.00	1.00	1.00
	PC	3	100%	100%	88.3%	0.65	1.00	0.81	0.91	0.83

**Table 4:** Statistics of the number of classified pixels for the three meat roll classes.

Pixels in Different Classes	Raw Meat Rolls			Cooked Meat Rolls		
	Mutton	Pork	Duck	Mutton	Pork	Duck
Percentage of mutton pixels	79.87%	6.15%	13.98%	75.94%	4.70%	19.36%
Percentage of pork pixels	12.33%	57.99%	29.68%	19.31%	75.01%	5.68%
Percentage of duck pixels	13.51%	10.42%	76.07%	13.98%	0.16%	85.86%

## Data Availability

The data presented in this study are available on request from the corresponding author.
